# Giant extra-hepatic thrombosed portal vein aneurysm: a case report and review of the literature

**DOI:** 10.1186/1749-7922-9-35

**Published:** 2014-04-29

**Authors:** Ismaïl Labgaa, Yann Lachenal, Pierre Allemann, Nicolas Demartines, Markus Schäfer

**Affiliations:** 1 Department of Visceral Surgery, CHUV University Hospital, Rue du Bugnon 46, CH-1011, Lausanne, Switzerland; 2 Department of Radiology and Interventional Radiology, CHUV University Hospital, Lausanne, Switzerland

**Keywords:** Aneurysm, Thrombosis, Portal vein

## Abstract

**Background:**

Extrahepatic Portal vein aneurysm (EPVA) is a rare finding that may be associated with different complications, e.g. thrombosis, rupture, portal hypertension and compression of adjacent structures. It is being diagnosed more frequently with the advent of modern cross-sectional imaging. Our review of the English literature disclosed 13 cases of thrombosed EPVA.

**Case presentation:**

A 50-years-old woman presented with acute abdominal pain but no other symptom. She had no relevant medical history. Palpation of the right upper quadrant showed tenderness. Laboratory tests were unremarkable. A computed tomography showed portal vein aneurysm measuring 88 × 65 mm with thrombosis extending to the superior mesenteric and splenic vein. The patient was treated conservatively with anticoagulation therapy. She was released after two weeks and followed on an outpatient basis. At two months, she reported decreased abdominal pain and her physical examination was normal. A computed tomography was performed showing a decreased thrombosis size and extent, measuring 80 × 55 mm.

**Conclusions:**

Although rare, surgeons should be made aware of this entity. Complications are various. Conservative therapy should be chosen in first intent in most cases. We reported the case of the second largest thrombosed extra-hepatic PVA described in the literature, treated by anticoagulation therapy with a good clinical and radiological response.

## Background

Portal vein aneurysm (PVA) is defined as a focal dilatation of the portal venous system, greater than 2 cm [1]. PVA is a rare vascular anomaly, observed in 0.43% [2] but its incidence was increasing in recent years with the enlarged use of magnetic resonance (MR) and computed tomography (CT) [3]. Most common sites are the main portal vein and confluence of splenic and superior mesenteric veins, forming extra-hepatic portal vein aneurysm (EPVA). Although risk factors like portal hypertension and liver cirrhosis have been highlighted, the etiology remains to be clarified. PVA may be associated with various complications: thrombosis, aneurismal rupture, inferior vena cava obstruction and duodenal compression. Thrombosis is the most frequent complication with complete thrombosis and non-occlusive thrombus occurring in 13.6% and 6%, respectively [3]. Herein we report the case of a giant EPVA with complete thrombosis, among the largest described so far. A conservative treatment showed satisfying clinical and radiological response. We reviewed the English literature, disclosing 13 cases of thrombosed EPVA in order to assess current treatment [4-13].

## Case presentation

**Figure 1:**
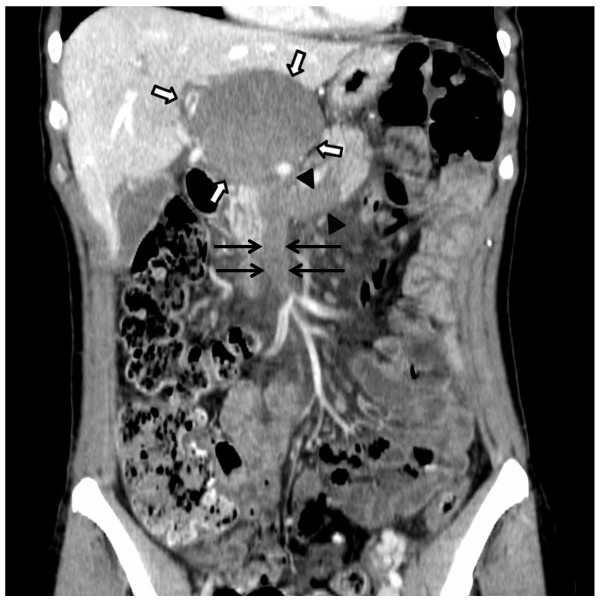
CT-scan showing thrombosed portal vein aneurysm (white arrows) with thrombus extending to SMV (black arrows) and splenic vein (arrowheads).

**Figure 2:**
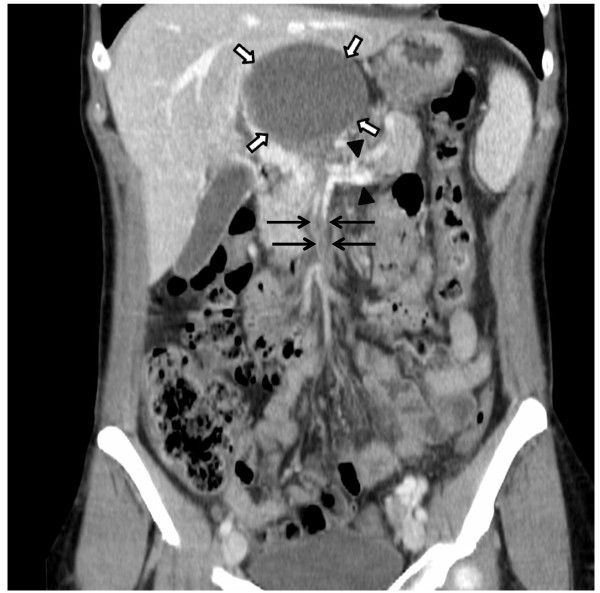
CT-scan showing decreasing size of thrombus within portal vein aneurysm (white arrows) with diminished extension to SMV (black arrows) and SV (arrowheads).

A 50-years old woman with no relevant medical history and no underlying liver disease was referred to our Division for acute abdominal pain but no other abdominal or systemic symptom. Palpation of the right upper quadrant showed tenderness but Murphy’s sign was negative. Lab tests showed slightly increased serum CRP (53 mg/L), normal white cell count, undisturbed coagulation blood tests, and liver function remained unremarkable. Tumor markers CA 19–9 and CEA were also normal, 3 kU/L and 1.1 ug/L, respectively. A CT showed portal vein aneurysm measuring 88 × 65 mm with complete thrombosis extending to superior mesenteric (SMV) and splenic (SV) veins (Figure 1). The risk of rupture being low, we decided to treat conservatively with anticoagulation therapy. We completed our investigations with an upper GI endoscopy and thrombophilia workup; the former did not show any esophageal varices indicating portal hypertension, and any coagulation disorder could be detected. The patient was released after two weeks and followed on an outpatient basis. At two months, she reported decreased pain, and a control CT demonstrated the decreasing of the thrombosis, measuring 80 × 55 mm, associated with a diminished extension to superior mesenteric and splenic veins (Figure 2).

## Discussion

Venous aneurysms remain much less common than arterial ones. The most common location for visceral venous aneurysms is portal system with almost 200 reported cases [3]. Notwithstanding PVA incidence has increased during the last decades, very probably due to the widened use of modern imaging techniques like MR and CT scans. Most frequent sites are the main portal vein and the SV-SMV confluence. The mechanisms and etiologies are not well understood but appeared to be acquired or congenital. Concerning the former, portal hypertension and chronic liver disease were identified as risk factors [8,14]. Other causes like pancreatitis, trauma and previous surgery were described as triggers [15-17]. Nevertheless, a significant number of PVA cases did not present any underlying liver disease; and embryological mechanisms causing PVA have been mentioned. The failure of complete regression of the right vitelline vein may be responsible for a venous saccular enlargement, leading to aneurysm. In our case, the patient did not present any risk factor: no underlying liver disease, no history of pancreatitis, trauma or abdominal surgery. These elements support the congenital cause. Hence, a genetic council was achieved and our workup was enlarged. Interestingly, the patient showed other anatomical variants: CT revealed four hepatic veins, and a lumbarization of S1 and a right supernumerary lumbar ribs, lateral to L1. Apparently, the patient was an isolated case with negative family history for anatomic anomalies.

PVA complications are various and thrombosis is the most frequent one. Patients with thrombophilia have a higher risk to develop portal vein thrombosis. In our case this cause was excluded. The review of the literature disclosed 13 cases of thrombosed EPVA [4-13]. The largest one, measuring 81 × 109 mm was reported by *Oleske A and Hines GL*[4] and was also successfully treated conservatively.

The level of evidence regarding the management of thrombosed EPVA remains low as only few cases have been published so far. Nevertheless, authors considered clinically symptomatic patients and complete thrombosis of PVA as indications for surgery [7,9,18]. Brock et al. postulated that patients with thrombosis extending to SMV and SV should undergo thrombectomy and restoration of portal vein anatomy [19]; but complication rates of surgical management have not been reported. It can be strongly assumed that a conservative treatment has lower complication rates, and reported conservative treatments of thrombosed EPVA have provided good results, as in our case [5,8,10,12]. Subsequently, we would not consider presence of symptoms or thrombosis as strict indications for surgery, and a conservative approach and follow-up in first intent even for aneurysm of great size or extension to SMV/SV is recommended. This approach is also supported by the low risk of aneurismal rupture, 2.2% [3]. In case of treatment failure, surgical treatment should be considered.

## Conclusions

Although rare PVA are being more and more frequent. General surgeons should be made aware of this entity, taking part in a differential diagnosis of abdominal pain. Mechanisms and etiologies remain ill defined. We report the case of the second largest extra-hepatic portal vein aneurysm with complete thrombosis, described so far. The patient was treated conservatively with good clinical and radiological response. This case supports a conservative strategy for PVA, in first intent.

### Consent

Written informed consent was obtained from the patient for publication of this Case report and any accompanying images. A copy of the written consent is available for review by the Editor-in-Chief of this journal.

## Abbreviations

PVA: Portal vein aneurysm; EPVA: Extra-hepatic portal vein aneurysm; CT: Computed tomography; CRP: C-Reactive protein; US: Ultrasound; MR: Magnetic resonance; SV: Splenic vein; SMV: Superior mesenteric vein.

## Competing interests

The authors who have taken part in this case report declared that they do not have anything to disclose regarding funding or conflict of interest with respect to this manuscript.

## Authors’ contributions

Labgaa I and Schäfer M designed the report; Labgaa I, Allemann P and Schäfer M were the attending doctors for the patient; Lachenal Y provided radiological pictures; Labgaa I wrote the paper; Allemann P, Demartines N and Schäfer M reviewed the paper. All authors read and approved the final manuscript.
